# Primary cardiac sarcomas: A clinicopathologic study in a single institution with 25 years of experience with an emphasis on MDM2 expression and adjuvant therapy for prognosis

**DOI:** 10.1002/cam4.6303

**Published:** 2023-07-03

**Authors:** Haeyon Cho, In‐Hye Song, Uiree Jo, Ji‐Seon Jeong, Hyun Jung Koo, Dong Hyun Yang, Sung‐Ho Jung, Joon Seon Song, Kyung‐Ja Cho

**Affiliations:** ^1^ Department of Pathology University of Ulsan College of Medicine, Asan Medical Center Seoul Republic of Korea; ^2^ Department of Radiology and Research Institute of Radiology University of Ulsan College of Medicine, Asan Medical Center Seoul Republic of Korea; ^3^ Department of Thoracic and Cardiovascular Surgery University of Ulsan College of Medicine, Asan Medical Center Seoul Republic of Korea

**Keywords:** cardiac sarcoma, heart transplantation, intimal sarcoma, MDM2, survival

## Abstract

**Background:**

Primary cardiac sarcomas are rare and their clinicopathologic features are heterogeneous. Among them, particularly intimal sarcoma is a diagnostic challenge due to nonspecific histologic features. Recently, MDM2 amplification reported to be a characteristic genetic event in the intimal sarcoma. In this study, we aimed to identify the types and incidence of primary cardiac sarcomas that occurred over 25 years in tertiary medical institutions, and to find clinicopatholgical significance through reclassification of diagnoses using additional immunohistochemistry (IHC).

**Methods:**

We reviewed the primary cardiac sarcoma cases between January 1993 and June 2018 at Asan Medical Center, South Korea, with their clinicopathologic findings, and reclassified the subtypes, especially using IHC for MDM2 and then, analyzed the significance of prognosis.

**Results:**

Forty‐eight (6.8%) cases of a primary cardiac sarcoma were retrieved. The tumors most frequently involved the right atrium (*n* = 25, 52.1%), and the most frequent tumor subtype was angiosarcoma (*n* = 23, 47.9%). Seven cases (53.8%) were newly reclassified as an intimal sarcoma by IHC for MDM2. Twenty‐nine (60.4%) patients died of disease (mean, 19.8 months). Four patients underwent a heart transplantation and had a median survival of 26.8 months. This transplantation group tended to show good clinical outcomes in the earlier stages, but this was not statistically significant (*p* = 0.318). MDM2 positive intimal sarcoma showed the better overall survival (*p* = 0.003) than undifferentiated pleomorphic sarcoma. Adjuvant treatment is beneficial for patient survival (*p* < 0.001), particularly in angiosarcoma (*p* < 0.001), but not in intimal sarcoma (*p* = 0.154).

**Conclusion:**

Our study supports the use of adjuvant treatment in primary cardiac sarcoma, as it was associated with a significantly better overall survival rate. Further consideration of tumor histology may be important in determining the optimal use of adjuvant treatment for different types of sarcomas. Therefore, accurate diagnosis by MDM2 test is important condsidering patient's prognosis and treatment.

## 1 INTRODUCTION

Primary cardiac tumors are rare and the incidence in the early autopsy series ranged from 0.001% to 0.3%.^1^ Approximately 75% of primary cardiac tumors are benign such as cardiac myxomas, with the remaining 25% of showing malignancy, the majority of which are is sarcomas.^2^ Most cardiac sarcomas have been published as single case reports or institutional small series, with only a few studies examining large sample sizes.^3^, ^4^

Primary cardiac sarcomas represent a heterogenous group of tumors, the most common of which are angiosarcomas,^3^ followed by intimal sarcomas.^5^ Notably, the current classification of intimal sarcoma is still controversial.^5^, ^6^

Mouse double minute 2 *(MDM2)* is a critical negative regulator of the tumor suppressor *p53*, playing a key role in controlling its transcriptional activity, protein, stability, and nuclear localization. *MDM 2* expression is upregulated in numerous cancers, resulting in a loss of *p53*‐dependent activities, such as apoptosis and cell cycle arrest.^7^ MDM2 amplification is found in many subtypes of sarcomas including osteosarcoma, well differentiated/ dedifferentiated liposarcoma (DDLPS), and rhabdomyosarcoma.^8^, ^9^, ^10^ In addition, *MDM2* amplification was shown to associated with overexpression of both RNA and protein.^11^, ^12^ Among major sarcomas of large vessels, intimal sarcoma has been firstly describled overexpression and amplification of *MDM2* ^13^ in 2001.

Primary cardiac sarcomas show a dismal prognosis. Yin et al. reported a median survival of only 7 months for affected patients, and 1‐, 3‐, and 5‐year survival rates of 40.7%, 15.6%, and 9.8%.^3^ Complete surgical excision and adjuvant chemotherapy are associated with increased survival.^3^

Much of the previously published data on cardiac tumors do not reflect the current updated disease classifications or entity description that have arisen from improved molecular and immunohistochemical diagnostic methods. Studies by clinicians based only on past medical records without a pathologist's review are also likely to produce erroneous data. Herein, we have reviewed the primary cardiac sarcoma patients at our hospital over a 25 year periods and classified the tumors in each cases in accordance with recent updates to these disease entities using additional immunohistochemistry (IHC). We also evaluated the clinicopathologic significance of these new evaluations.

## 2 MATERIALS AND METHODS

### 2.1 Study design

We retrospectively reviewed 705 patients who underwent cardiac surgery for cardiac tumors between January 1993 and March 2018 at Asan Medical Center, Seoul, Korea. We identified 48 patients (6.8%) with a primary cardiac sarcoma. All of the study cases were histologically and radiologically reviewed by two bone and soft tissue pathologists (J.S.S and K.J.C) and two radiologists (H.J.K, and D.H.Y) along with their pathologic diagnosis, IHC, clinical data, and medical records. The Institutional Review Board of Asan Medical Center approved our study protocol.

### 2.2 Immunohistochemistry

IHC was newly performed to confirm the original diagnosis. Whole tissue was cut into 4‐μm thick sections. The IHC was performed using the Ventana NX automated immuno‐histochemistry system (Ventana Medical systems). The retrieved antibodies were as follows: SMA (1:200; Dako), MDM2 (1:100, NeoMarkers), Desmin (1:200, Dako), CD31(1:100, Novocastra Labratories Ltd), CD34 (1:500, Immunotech), CD99 (1:200; Dako), S‐100 protein (1:200, Zymed), TLE‐1 (1:400, Novus), p53 (1:1500, Dako), NKX2.2 (1:400, clone 74.5A5, DSHB), and myogenin (1:200, NeoMarkers). After incubation with primary antibodies, the sections were incubated with an UltraView universal DAB kit (Ventana Medical System).

### 2.3 Statistical analysis

Survival analysis was performed using the Kaplan–Meier method and the resulting curves were compared using the log‐rank test. The results were considered statistically significant when the *p*‐value was <0.05. All statistical analyses were carried out using the SPSS software package, version 21.0 (SPSS).

## 3 RESULTS

### 3.1 Clinicopathologic findings

Forty‐eight patients (6.8%) with primary cardiac sarcoma out of 705 patients for cardiac tumor were retrieved. There were 21 men and 27 women with mean age of a 47.2 years (range, 12–78 years). The most common presenting symptom was dyspnea with a New York Heart Association functional classification of greater than II (*n* = 22, 45.8%) and the second was chest pain or chest discomfort (*n* = 8, 16.7%). Other symptoms include chronic cough (*n* = 6, 12.5%), syncope (*n* = 3, 6.2%), pericardial effusion (*n* = 4, 8.3%) and incidental findings during a regular medical check‐up (*n* = 2, 4.2%).

**FIGURE. 1:**
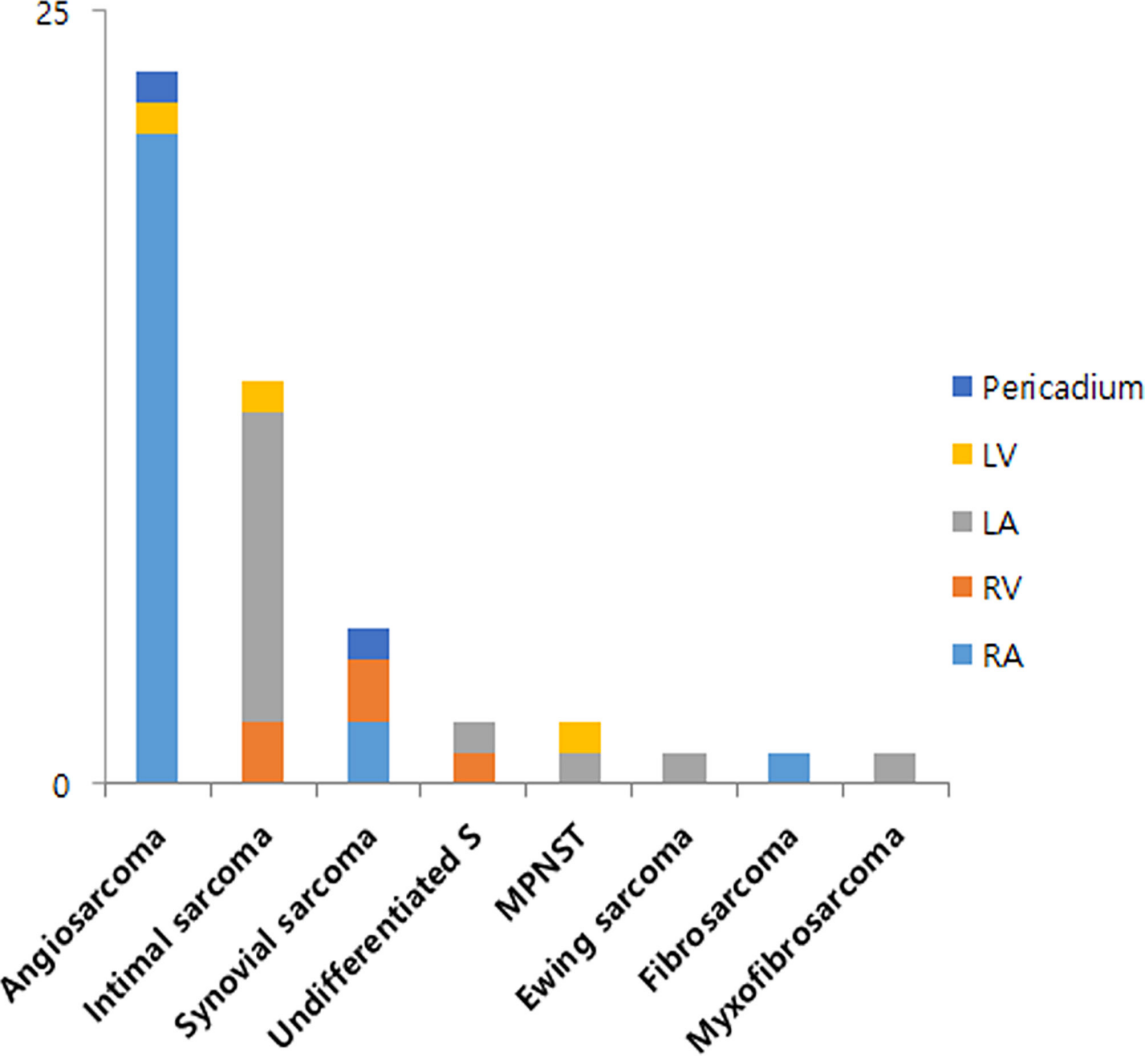
Incidence of primary cardiac sarcoma by location. The most frequent histologic type is an angiosarcoma (*n* = 23, 47.9%) following by an intimal sarcoma (*n* = 13, 27.1%). In 21 (91.3%) cases with an angiosarcoma, the lesions arise in the right atrium (RA), while 11 (84.6%) of the intimal sarcomas are located in the left atrium (LA). LV, left ventricle; MPNST, malignant peripheral nerve sheath tumor; RV, right ventricle; Undifferentiated S., Undifferentiated pleomorphic sarcoma.

The tumor most frequently involved the right atrium (*n* = 25, 52.0%), followed by the left atrium (*n* = 15, 31.2%) and a specific tumor histology was associated with these different sites. The histologic types were angiosarcoma (*n* = 23, 47.9%), intimal sarcoma (*n* = 13, 27.1%), synovial sarcoma (*n* = 5, 10.4%), undifferentiated pleomorphic sarcoma (UPS, *n* = 2, 4.2%), myxofibrosarcoma (*n* = 1, 2.1%), fibrosarcoma (*n* = 1, 2.1%), malignant peripheral nerve sheath tumor (MPNST, *n* = 2, 4.2%) and Ewing sarcoma/primitive neuroectodermal tumor (ES/PNET, *n* = 1, 2.1%) (Figure 1A). Twenty‐one (91.3%) of the 23 angiosarcomas originated from the right atrium with one each occurring in the left ventricle and pericardium. The most common site of intimal sarcomas originated from the left atrium (*n* = 11, 84.6%) (Figure 1B). The clinicopathologic characteristics are summarized in Table 1 and Table S1.

**TABLE. 1:** Clinical and pathologic characteristics of cardiac primary sarcoma.

No	Sex	Age	Presenting symptom	Initial Dx	Revised Dx	Site	OP title	Neoadjuva‐nt Tx	Adjuvant Tx	Recurrence/Metastasis (m)	Outcomes (m)	Ancillary test
1	M	39	Dyspnea	AS	AS	RA	Incomplete Ex	Unknown	No	Brain (12d)	DOD (0.5)	CD34 (+)
2	M	20	Chest pain	AS	AS	RA, RV	Pericardiotomy	No	CTx & RTx	Lung (8.8)	DOD (12.8)	CD31 (+)
3	M	68	Pericardial effusion	AS	AS	RA	Incomplete Ex	No	No	No	DOD (1d)	CD31 (+)
4	M	51	Arrhythmia	AS	AS	RA	Bx	unknown	Unknown	Liver (3.6)	DOD (17.9)	CD31 (+), CD34 (+)
5	F	28	Pericardial effusion	AS	AS	RA	Complete Ex	No	RTx	No	DOD (12.4)	CD31 (+), CD34 (+)
6	M	34	Chest pain	AS	AS	PC	Bx	No	CTx	No	DOD (17.6)	CD31 (+), CD34 (+)
7	M	72	Chest discomfort	AS	AS	RA	Bx	No	No	No	DOD (0.5)	CD31 (+), CD34 (+)
8	M	23	Syncope	AS	AS	RA	Complete Ex	No	CTx	Pleura (31)	DOD (38.4)	CD31 (+), CD34 (+)
9	M	31	Dyspnea	AS	AS	RA	Complete Ex	No	CTx	Liver & Spleen (6.9)	DOD (14.9)	CD31 (+), CD34 (+)
10	F	37	Syncope	AS	AS	RA	Incomplete Ex	No	RTx	No	DOD (6.3)	
11	F	60	Chronic chough	AS	AS	RA	Bx	No	CTx	No	NA (19.8)	CD31 (+), CD34 (+)
12	M	54	Chest discomfort	AS	AS	RA	Bx	No	CTx	No	NA (12.0)	
13	M	39	Dyspnea	AS	AS	RA	Bx	No	CTx	Liver	DOD (9.6)	CD31 (+), CD34 (+)
14	F	66	Medical check‐up	AS	AS	RA	HTPL	No	CTx	Neck (20)	DOD (23.6)	
15	M	43	Chest pain	AS	AS	RA	Complete Ex	No	CTx & RTx	No	DOD (20)	CD31 (+), CD34 (+)
16	F	61	Medical check‐up	AS	AS	LV	Complete Ex	No	CTx	No	Alive (78)	CD31 (+), CD34 (+)
17	F	71	Pericardial effusion	AS	AS	RA	Incomplete Ex	No	CTx	No	DOD (6.0)	NA
18	M	53	Dyspnea	AS	AS	RA	Incomplete Ex	No	CTx & RTx	Gingiva & scapula (19.7)	NA(40.0)	CD31 (+), CD34 (+)
19	F	35	Chest pain	AS	AS	RA	Incomplete Ex	No	CTX	Heart (19.0)	NA (39.0)	NA
20	M	63	Dyspnea	AS	AS	RA	Incomplete Ex	No	CTX	Liver & bone (21.0)	DOD (29.0)	CD31 (+), CD34 (+)
21	F	56	Dyspnea	AS	AS	RA	Incomplete Ex	CTX & RTx	CTx	Liver (10)	NA (25.0)	NA
22	F	56	Syncope	AS	AS	RA	Complete Ex	CTX	CTx & RTx	Liver (9.3)	NA (10.0)	NA
23	M	37	Dyspnea	AS	AS	RA	Incomplete Ex	No	CTX	Lung (7.2)	DOD (10.0)	NA
24	F	29	Dyspnea	ES/PNET	ES/ PNET	LA	HTPL	CTx	CTx & RTx	Lung (12.5)	DOD (40.0)	CD99 (+), NKX2.2 (+)
25	F	12	Abdominal pain	FS	FS	RA, RV	Pericardiotomy	CTx	CTx & RTx	Liver (87.7)	NA (87.7)	MDM2 (−)
26	M	60	Dyspnea	MS	IS	LA	Incomplete Ex	No	CTx	Skull & Esophagus (3.8)	DOD (4.8)	MDM2 (+), P53 (+)
27	M	51	Dyspnea	MFH	IS	LA	O&C	No	CTx	Brain (9.5)	DOD (10.5)	MDM2 (+)
28	M	73	Dyspnea	MFH	IS	LA	Incomplete Ex	No	No	No	DOD (1.2)	MDM2 (+), P53 (+)
29	F	77	Chronic cough	MFH	IS	LA	Incomplete Ex	No	No	No	DOD (2.2)	MDM2 (+)
30	F	58	Chronic cough	MS	IS	LA	Complete Ex	No	CTx & RTx	LA (6.5)	DOD (13.5)	MDM2 (+), P53 (+)
31	F	34	Chest pain	LMS	IS	LA, LV	HTPL	RTx	CTx	Pancreas (12)	DOD (34.9)	MDM2 (+)
32	F	78	Dyspnea	IS	IS	LA	Complete Ex	No	No	No	NA (1.9)	MDM2 (+)
33	M	37	Dyspnea	IS	IS	RV	Incomplete Ex	No	CTx	No	NA (7.2)	MDM2 (+)
34	F	44	Dyspnea	MFH	IS	LA	Incomplete Ex	Unknown	CTx	Lung (16.0)	NA (16.0)	MDM2 (+)
35	M	17	Dyspnea	IS	IS (OSA)	LA	HTPL	No	No	No	DOD(9.0)	MDM2 (+), P53 (+)
36	F	56	Dyspnea	IS	IS	LA	Incomplete Ex	No	No	LA (4) & Rt. pulmonary vein (18.7)	DOD (25.9)	MDM2 (+), P53 (+)
37	M	71	Dyspnea	IS	IS	RVOT	Complete Ex	No	CTx	Lung & brain (7.7)	DOD (10.4)	NA
38	F	42	Cough & dyspnea	IS	IS	LA	Incomplete Ex	No	CTx	Liver (0.5)	Alive (65.1)	MDM2 (+), P53 (+)
39	F	32	Chest pain	MPNST	MPNST	LV	Complete Ex	No	CTx	Lung (12.2)	DOD (48.2)	S‐100 (focal +)
40	F	50	Chest pain	MPNST	MPNST	LA	Incomplete Ex	No	CTx & RTx	Bone (9.0)	NA (13.0)	S‐100 (focal +), NESTIN (+), MDM2 (+)
41	F	36	Dyspnea	MFS	MFS	LA	Incomplete Ex	No	CTx & RTx	Brain (?)	DOD (14.0)	MDM2(+/−), P53(+)
42	F	16	Dyspnea	SS	SS	RA, RV	Incomplete Ex	Unknown	CTx	No	NA (85.1)	
43	F	58	Dysphagia	SS	SS	RA	Incomplete Ex	No	No	No	DOD (6.1)	SYT‐SSX translocation (+)
44	F	34	Chronic cough	SS	SS	PC	Complete Ex	No	CTx	No	NA (23.2)	TLE‐1 (+)
45	M	39	Pericardial Effusion	SS	SS	RV	Incomplete Ex	No	CTx	Pericardium (41.0)	Alive (65.0)	CK (focal +)
46	M	66	Dyspnea	SS	SS	RA, RV	Incomplete Ex	No	Unknown	Unknown	NA(0.5)	TLE‐1 (+), CK (+)
47	F	27	Chest pain and dyspnea	E‐RMS	UDS	LA	Incomplete Ex	No	No	No	DOD (0.5)	MDM2 (−), desmin (−), myogenin (−)
48	F	76	Chronic cough	UDS	UDS	RV	Bx	Unknown	No	Unknown	DOD (1.3)	MDM2 (−)

Abbreviations: AS, angiosarcoma; AWD, alive with disease; Bx, biopsy; CTx, chemotherapy; DOD, die of disease; Dx, diagnosis; Ex, excision; E‐RMS, embryonal rhabdomyosarcoma; ES/PNET, ewing sarcoma/ primitive neuroectodermal tumor; F, female; FS, fibrosarcoma; HTPL, heart transplantation; IHC, immunohistochemistry; IS, intimal sarcoma; LMS, leiomyosarcoma; LA, left atrium; LV, left ventricle; M, male; MS, Myxosarcoma; MFS, Mysofibrosarcoma; MPNST, malignant pheripheral nerve sheath tumor; NA, not accessible; OP, operation; PC, pericardium; RTx, Radiation therapy; RA, right atrium; RV, right ventricle; SS, synovial sarcoma; Tx, treatment; UPS, undifferentiated pleomorphic sarcoma.

We performed IHC for MDM2 and p53 to confirm the diagnosis in 14 cases which were initially diagnosed as angiosarcoma, MPNST, ES/PNET, and synovial sarcoma. Seven cases were reclassified as an intimal sarcoma in accordance with their MDM2 immunopositivity. Two of these cases had been initially diagnosed as myxosarcomas, four as a malignant fibrous histiocytoma and one as a leiomyosarcoma. Patient 47 was initially diagnosed with an embryonal rhabdomyosarcoma but was found in our current analyses to be immunonegative for all of the tested muscle markers including desmin, myogenin, myoglobin, and myoD1. Histologically, the tumor cells in this case were composed of short spindle cells with a short fascicular pattern and alternating cellularity. They did not show any specific lineage by IHC or ultrastructural examination, and the final diagnosis was revised to an undifferentiated spindle cell sarcoma.

**FIGURE 2:**
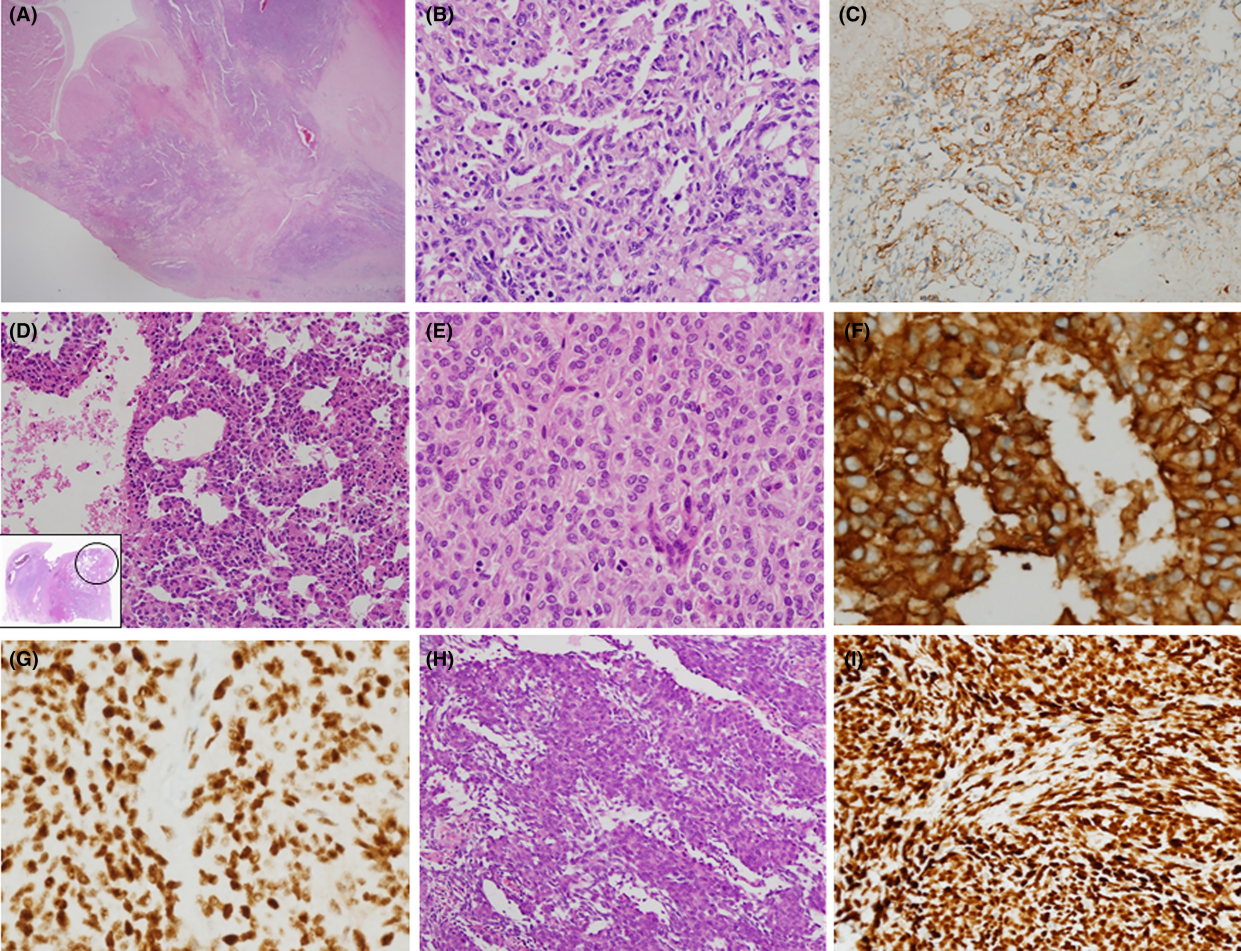
Pathologic features for angiosarcoma (A–C), Ewing sarcoma/PNET (D–G) and synovial sarcoma (H–I). (A) Mass infiltrating the right atrial wall with irregular margins. (B) Slit–like irregularly anastomosing vessel patterns were noted, lined with atypical, hyperchromatic cells with numerous mitotic activity. (C) The atypical cells were positive for CD31 by immunohistochemistry. (D) A 29 years old woman received neoadjuvant chemotherapy for a left atrial mass and a heart transplantation was performed. Epithelioid cells with ectatic vessels were noted (o, inset) mimicking angiosarcoma. Atypical round cells with eosinophilic cytoplasm (E) were positive for CD99 (membranous pattern, (F) and NKX 2.2 (G) by immunohistochemistry, supporting the diagnosis of a Ewing sarcoma. (H) A 34 years old woman underwent an excision for a pericardial mass. The mass comprised short spindle cells and epithelioid cells with high cellularity. (I) The tumor cells were positive for TLE‐1 by immunohistochemistry, supporting the diagnosis of a synovial sarcoma.

Representative pathologic features of sarcomas including angiosarcoma (Figure 2A–C), Ewing sarcoma (Figure 2D–G) and synovial sarcoma (Figure 2H,I) were shown in Figure 2.

**FIGURE 3:**
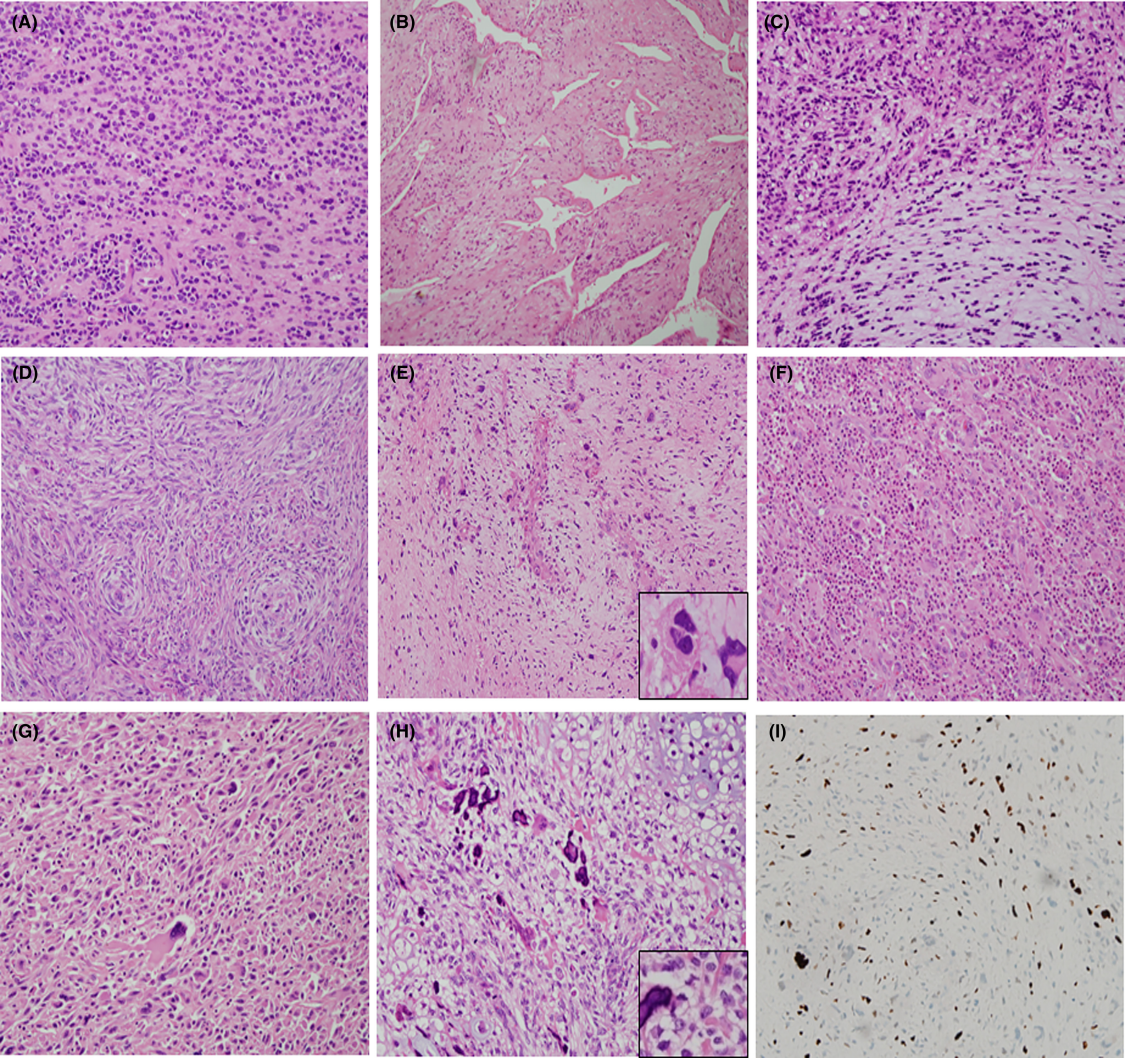
Various histologic features of the intimal sarcomas. (A) An Epithelioid and round cell sarcoma –like pattern (patient 23). (B) A hemangiopericytoma‐like pattern (patient 19). (C) Short spindle cells with a myxoid stroma (patient 25). (D) Spindle cells with a storiform pattern (patient 24). (E) Mixed spindle cells with atypical pleomorphic cells (inset) in a myxoid stroma with a thick‐walled branching vasculature (patient 23). (F) Pleomorphic multinucleated giant cells in a background of inflammatory cells (patient 27). (G) Pleomorphic cells with rhabdomyosarcomatous differentiation (patient 22). (H) Osteosarcomatous differentiation (patient 28). (I) All of the intimal sarcoma patients showed immunopositivity for MDM2 and representative images are shown (patient 23).

The intimal sarcomas in our present series showed variable histologic features including a spindle cell sarcoma with myxoid stroma, hemangiopericytoma‐like sarcoma, osteosarcomatous differentiation, a pleomorphic sarcoma with extensive inflammatory infiltrates, and a mixed epithelioid, spindle, myxoid, and pleomorphic sarcoma pattern (Figure 3A–H). All of these tumors were positive for MDM2 by IHC (Figure 3I).

**FIGURE 4:**
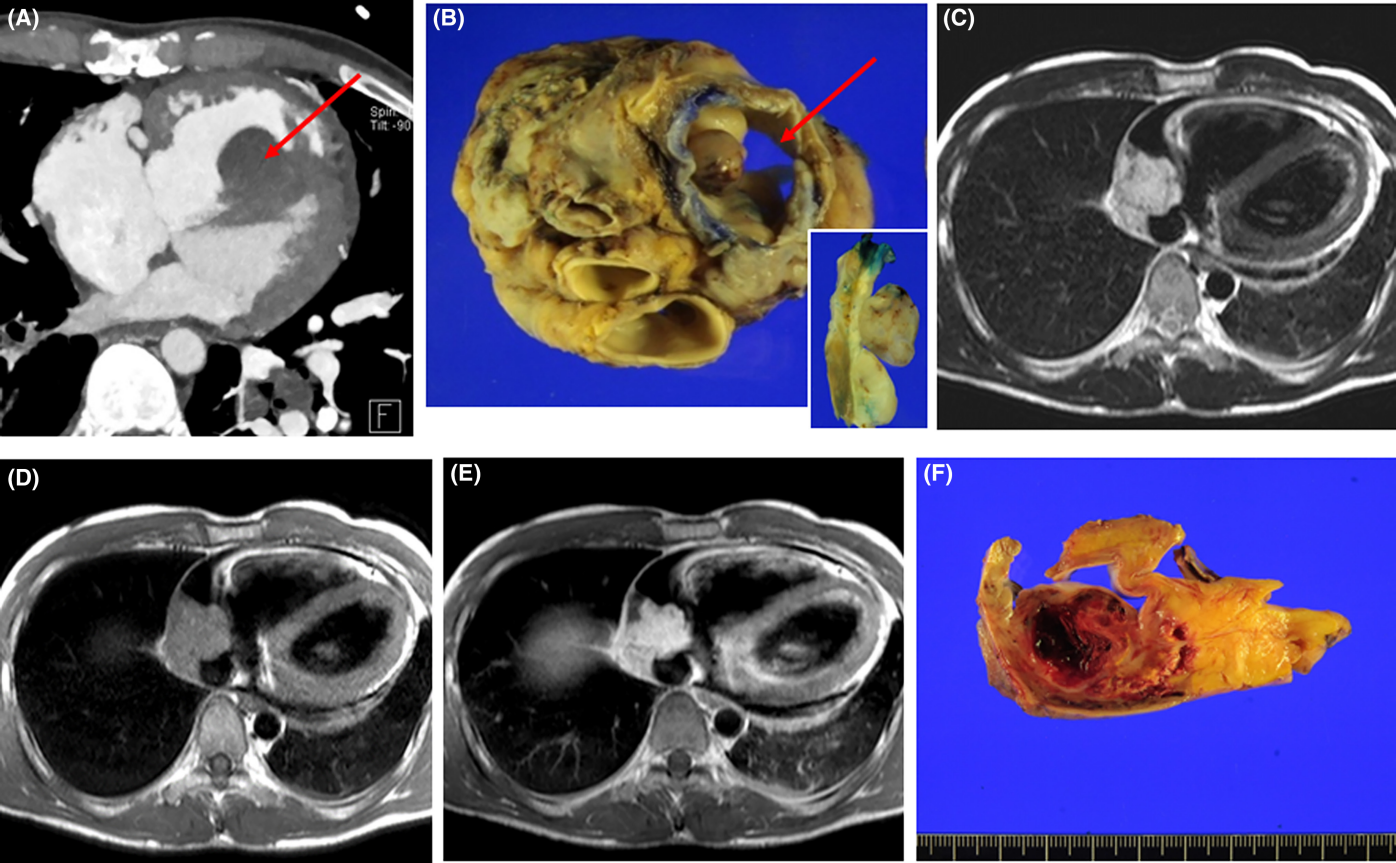
Radiologic and gross findings for the intimal sarcoma (A–B) and angiosarcoma (C–F) patients treated with a heart transplantation. (A, B). A 17 years‐old man received a heart transplantation due to a left atrial mass. A cardiac CT (A) showed a non‐enhanced mass (red arrow) with a suspicion of a focal myocardial invasion. The patient underwent a heart transplantation, and the polypoid mass was found to have been present in the endocardium of the left atrium (B, red arrow). Grossly, the cut surface of the tumor was gray tan and fibrotic without hemorrhage (inset). (C–F). A 66 years‐old woman underwent a heart transplantation due to a right atrial mass. Magnetic resonance images revealed that the mass (white arrow) was T2 high signal intensity (C), T1 low signal intensity (D) and T1 enhanced signal intensity (E), suggestive of an angiosarcoma. (F) Grossly, the mass filled the entire right atrium (RA) and had a hemorrhagic cut surface.

Eleven of patients underwent a complete excision, 23 received an incomplete excision, 10 had a biopsy or pericardiotomy, and four received a heart transplantation. Radiologic and gross findings for the intimal sarcoma (Figure 4A,B) and angiosarcoma (Figure 4C–F) in heart transplantation cases were shown in Figure 4. Four of patients were administered neoadjuvant chemotherapy or radiation therapy and their diagnoses were angiosarcomas, fibrosarcoma and intimal sarcoma, respectively. Thirty‐four patients received adjuvant chemotherapy, radiation therapy or chemoradiation therapy.

### 3.2 Correlation between MDM2 expression and clinicopathologic parameters

The testing for MDM2 expression is mainly done to distinguish intimal sarcoma from UPS. A total of 15 patients were included in the analysis, and all MDM2 positive patients were classified as intimal sarcoma. These were summarized in Table 2. MDM2 positivity was correlated with tumor location, especially left atrium (*p* = 0.001), which is consistent with the common occurrence of intimal sarcoma at that location.

**TABLE 2:** Correlation between MDM2 expression and clinicopathologic parameters.

Parameters	MDM2 positive (*N* = 13, %)	MDM2 negative (*N* = 2, %)	*p* Value^a^
Age (median, years)	56 (range, 17–78)	51 (range, 17–76)	0.378
Gender			0.486
Male	6 (40.0%)	0 (0%)	
Female	7(46.7%)	2 (13.3%)	
Histology			
Intimal sarcoma	13 (86.7%)	0 (0%)	<0.001
UPS	0 (0%)	2 (13.3%)	
Tumor location			0.001
Right atrium	0 (0%)	1 (6.7%)	
Left atrium	11 (73.3%)	0 (0%)	
Right ventricle	1 (6.7%)	1 (6.7%)	
Left ventricle	1 (6.7%)	0 (0%)	
Pericardium	0 (0%)	0 (0%)	
Surgical treatment			0.562
Incomplete excision	8 (53.3%)	2 (13.3%)	
Complete excision	3 (20.0%)	0 (0%)	
Heart transplatation	2 (13.3%)	0 (0%)	
Adjuvant Tx			0.267
No	5 (33.3%)	2 (13.3%)	
CTx	7 (46.7%)	0 (0%)	
RTx	0 (0%)	0 (0%)	
CTx + RTx	1 (6.7%)	0 (0%)	
Neoadjuvant Tx			0.773
Yes	1 (7.1%)	0 (0%)	
No	12 (85.7%)	1 (7.1%)	

^a^ Fisher's Exact test was performed due to small number of MDM2 negative patient.

### 3.3 Survival analysis

Follow‐up data were obtained in 34 of the study patients and the periods ranged from one day to 87.7 months (mean 15.9 months). Twenty‐nine (85.3%) patients died of disease (mean, 19.8 months) and five (14.7%) were alive with disease at the time of writing. Twenty‐seven patients (79.4%) underwent recurrence and/or metastasis, most commonly in the brain, lung, and liver.

**FIGURE 5:**
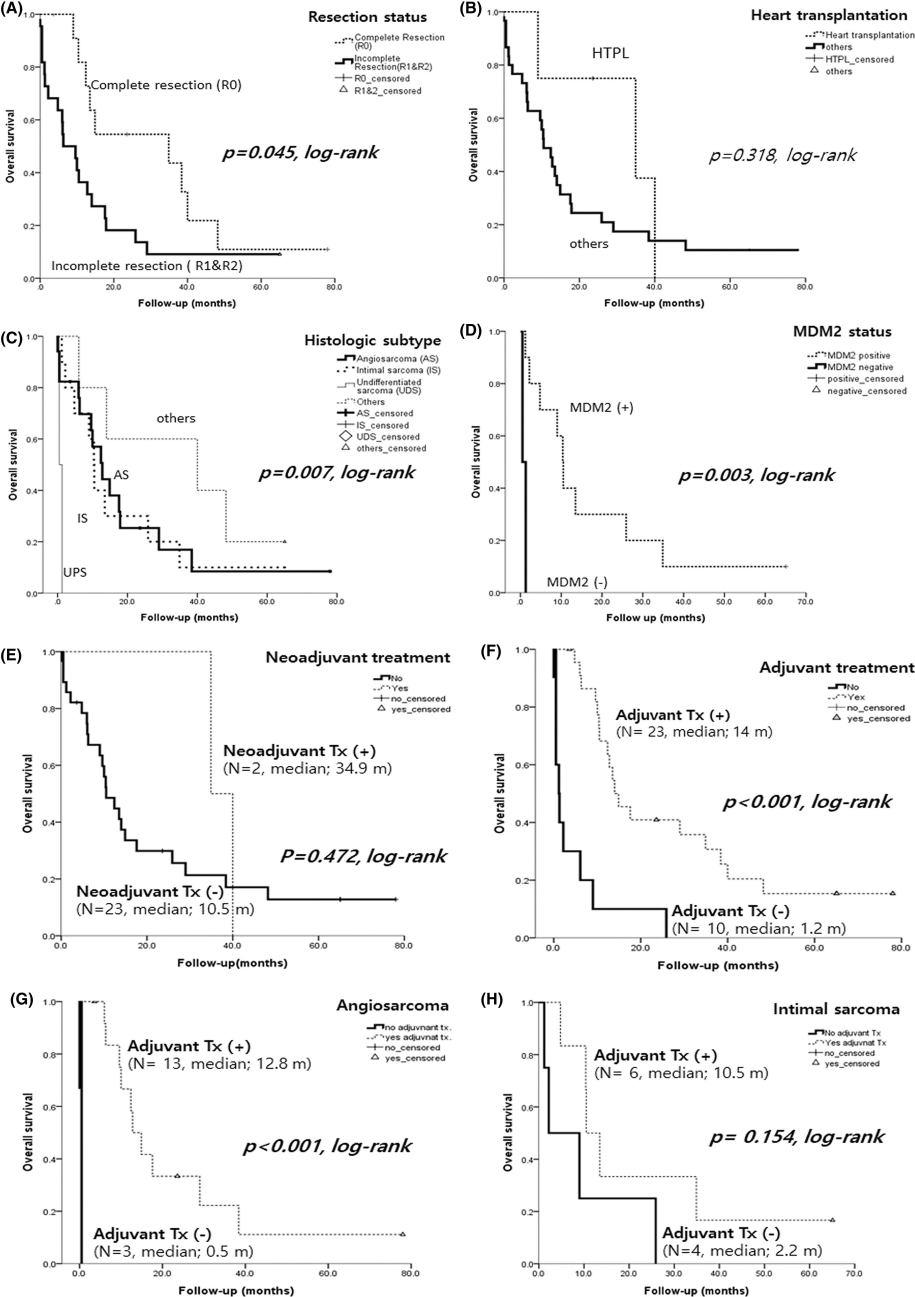
Kaplan–Meier survival analysis. (A) A complete excisions, including a heart transplantation, was associated with a better prognosis than an incomplete resection (*p* = 0.045, log‐rank). (B) Four patients underwent a heart transplantation (HTPL) and HTPL group appeared to have an improved clinical outcomes in the early stages, but no overall survival benefit was evident (*p* = 0.315, log‐rank). (C) The overall survival rates among histologic types were partly significant. No definite differences in these outcomes were found between angiosarcoma and intimal sarcoma (*p* = 0.664, data not shown) but patient s with an undifferentiated sarcoma (UPS) showed the poorest prognosis (*p* = 0.007, log‐rank). (D) MDM2 positive cases (intimal sarcoma) had better outcomes than MDM2 negative cases (UPS, *p* = 0.003, log‐rank), further stressing the importance of accurately diagnosing them. (E) The patient group that received neoadjuvant treatment did not show any significant benefit in overall survival (*p* = 0.472. log‐rank). (F) Adjuvant treated group showed better overall survival compared to non‐treated group (*p* < 0.001, log‐rank). The median survival time for the group that received adjuvant treatment was 14 months, whereas the group that did not receive treatment showed a difference with a median survival time of 1.2 months. (G) When restricting the analysis to anigosarcoma patients (*n* = 16), adjuvant treated group showed the better survival rate than non‐treated group (*p* < 0.001), suggesting the effectiveness of postoperative radiotherapy and chemotherapy. However, when restricting the analysis to intimal sarcoma patients (*n* = 10, H), the patients who received adjuvant treatment did not show any significant benefit in overall survival (*p* = 0.154).

A complete excision, including heart transplantation, produced a better prognosis than an incomplete resection (*p* = 0.045, log‐rank, Figure 5A). Four patients underwent a heart transplantation and this appeared to yield good clinical outcomes in the early stages but not an overall survival benefit (*p* = 0.315, log‐rank, Figure 5B). The median survival time in these four transplanted patients was 29.5 months (range, 9–40 months) and all died during follow‐up. Therefore, compared to patients who receive a heart transplantation for nonneoplastic cardiac disease, performing a heart transplantation on patients with primary cardiac sarcoma is not an effective treatment option.

The overall survival rates among histologic types were partly significant. No differences were found in the outcomes between angiosarcoma and intimal sarcoma (*p* = 0.664, data not shown), but an UPS had the poorest prognosis (*p* = 0.007, log‐rank, Figure 5C). Patients with MDM2 positive group had better outcomes than MDM2 negative cases (*p* = 0.003, log‐rank, Figure 5D), thus stressing the importance of accurately diagnosing these lesions and distinguishing them from undifferentiated sarcomas. There were no survival benefits found to be associated with the tumor location (not shown), or with the use of neoadjuvant (*p* = 0.472, log‐rank, Figure 5E). However, adjuvant treated group showed better overall survival compared to non‐treated group (*p* < 0.001, Figure 5F). Moreover, our analysis based on tumor histology demonstrated that adjuvant treatment was particularly effective in improving overall survival in angiosarcoma (*p* < 0.001, Figure 5G), but no statistically significant difference was observed in intimal sarcoma (*p* = 0.154, Figure 5H).

## 4 DISCUSSION

In our present study, 48 cases of primary cardiac sarcoma diagnosed at our tertiary single institute over a 25‐year period were reviewed and the clinicopathologic findings were analyzed. In brief, the most common sarcoma was an angiosarcoma, followed by an intimal sarcoma, and these lesions generally showed a specific anatomic location in accordance with their subtypes. The median survival period was 19.8 months for the entire cohort. The angiosarcoma and intimal sarcoma cases showed similar survival outcomes but patients with an undifferentiated sarcoma showed the poorest prognosis. Four patients in our current series underwent a heart transplantation but this produced no overall survival benefit.

Our recently published sarcoma data^14^ indicated an overall 5‐year survival rate of about 78.3% for patients with a soft tissue sarcoma, regardless of the tumor location. This rate was 82.9% if these lesions were in the extremities, but was less than 71.7% for a thoracic sarcoma. Yin et al.^3^ reported a median survival of 7 months for their entire cardiac sarcoma cohort (range, 1–19 months) and a 5‐year survival rate of 9.8%. The prognosis for an intimal sarcoma is known to be poor with a mean survival period ranging from only 5–9 months in the patients with aortic lesions and 13–18 months in patients with a pulmonary sarcoma.^13^, ^15^ Our present study revealed similar results with a mean survival time of 12.48 months. Surprisingly, only one of the patient in our current series was living after 65 months. According to previously published angiosarcoma data from our hospital,^16^ the mean overall survival for localized angiosarcoma was 21.6 months compared to only 12.56 months for primary cardiac angiosarcoma in our current study cases. Although the 5‐year survival rate for common angiosarcoma in our present series was about 40% regardless of the tumor location, there was no patient in our cohort with a primary cardiac angiosarcoma that survived past 5 years. Hence, in relation to sarcomas with the same histology, angiosarcomas of the heart seems to have a poorer prognosis than those at other sites.

Heart transplantation is an uncommon treatment for primary cardiac sarcoma and only 46 such cases had been reported by 2016 by Li et al.^17^ These authors reported that the overall median survival among cardiac sarcoma patients receiving a heart transplantation was 16 months (range, 2–112 months), but that the angiosarcoma cases in this group showed a poorer median survival time than this of 9 months. Our current study revealed that four patients received a heart transplant, including one angiosarcoma, two intimal sarcomas, and one Ewing sarcoma. The median survival time in these four study subjects was 29.5 months (range, 9–40 months). According to the current The International Society for Heart & Lung Transplantation (ISHLT) data,^18^ the median survival period after a heart transplantation is 13 years. Bhagra et al. described indication for cardiac transplantation,^19^and the authors classifies current and recent neoplasm as a contraindication for heart transplantation. For patients who have had malignant tumors in the past, individualized approaches are necessary. However, generally speaking, with the exception of non‐melanoma skin cancer, it is mostly contraindicated. In other words, it can only be attempted when the possibility of tumor recurrence and distant metastasis is very low. As mentioned before, while heart transplantation can extend lifespan by approximately 13 years in cases of nonneoplastic heart disease, the expected lifespan for hearts affected by cancer is only about 1 year. Therefore, in the case of primary heart sarcoma, it cannot be considered an appropriate treatment method due to cadaveric donor shortage. If advancements in science and technology lead to the widespread use of artificial hearts or animal hearts for transplantation, it may be reconsidered as a treatment option.

Intimal sarcomas are designated as follows by the 5th edition WHO classification of tumors: Soft tissue and bone tumors.^20^ These lesions are mesenchymal tumors arising in the large blood vessels of the systemic and pulmonary circulation, and also in the heart. Their defining features are predominantly an intraluminal growth with obstruction of the lumen of the vessel of origin, and the seeding of emboli to peripheral organs. The two essential criteria for diagnosing an intimal sarcoma are (1) occurrence within the lumen of a large vessel of the pulmonary or systemic circulation or within the heart cavities; and (2) primary high‐grade sarcoma, with or without heterologous elements. A desirable criterion for categorizing these tumors is *MDM2* gene amplification. In addition, WHO classification of tumors: Thoracic tumors 5th edition^21^ indicates that an intimal sarcoma belongs to the group of mesenchymal tumors specific to the lung, and designates it as a “pulmonary artery intimal sarcoma”. The WHO definitions for an intimal sarcoma are the same as those described in its soft tissue Bluebook. Notably however, in the cardiac tumor chapter, there is a disease entity termed “cardiac undifferentiated pleomorphic sarcoma (cardiac UPS)” instead of intimal sarcoma. In this WHO bluebook, the definition of cardiac UPS is a high‐grade sarcoma composed of pleomorphic spindled or epithelioid cells with no areas of differentiation that would indicate a s specific subset of sarcoma. Neuville et al^5^ suggested that the most frequent primary cardiac sarcoma is an intimal sarcoma based on the incidence of *MDM2 amplifications*. However, Maleszewski et al^6^ argued that cardiac sarcomas harboring an *MDM2 amplification* should not be referred to as intimal sarcoma because these gene amplifications are nonspecific and also occur in other sarcomas including well differentiated /DDLPS and synovial sarcoma. These authors also note that *MDM2 amplification* has been observed in 30% of cardiac UPS and in 70% of pulmonary artery intimal sarcomas. Hence, Maleszewski et al. have suggested the definition of a UPS with or without *MDM2* amplification instead of the term‐ intimal sarcoma in the heart.

Our current analyses revealed that intimal sarcomas exhibit a wide variety of histologic characteristics including myxoid, pleomorphic, inflammatory, myogenic, and osteosarcomatous features. Seven patients in our present cohort were given a revised diagnosis of intimal sarcoma in accordance with MDM2 immunopositivity. Recently, Yamada et al^22^ reported that a myxoid histology in an intimal sarcoma is associated with an *MDM2* amplification. There turned out to be no leiomyosarcomas among the primary cardiac sarcomas in our present study population. Although this was the initial diagnosis in one case, it was revised to an intimal sarcoma following our current MDM2 expression analysis. It is known that leiomyosarcomas account for about 20% of the total primary cardiac sarcoma cases and are mostly common located in the left atrium.^23^ Yamada et al^22^ and Neuville et al^5^ both revealed that 30% of intimal sarcomas are positive for smooth muscle actin, and show both myogenic differentiation and a comparable location profile to leiomyosarcoma. We anticipate therefore that a large number of intimal sarcomas will have been misclassified as leiomyosarcomas prior to the finding that *MDM2* amplification is a hallmark of these tumors. Notably in this regard, Neuville et al^5^ reported in a similar manner to our current study that of 12 cases in their sample population that had been initially classified as leiomyosarcoma, only two patients were confirmed with this diagnosis after a review.

The correlation between *MDM2* gene amplification in sarcoma and the clinical prognosis of the affected patient is controversial. A representative sarcoma that shows this amplification is DDLPS and a number of studies that have been principally related to prognosis have been conducted for this tumor subtype. Ricciotti et al^24^ reported that a high amplification of *MDM2* correlated with poor outcomes in DDLPS. Song et al^25^ revealed that both DDLPS and *MDM2* amplified UPS showed poorer overall survival times (39.5 months and 47.5 months, respectively) than *MDM2* non‐amplified UPS (61 months). Conversely however, Guellec et al^26^ described 2‐year overall survival rates for UPS with *MDM2* amplification, conventional DDLPS, and UPS without MDM2 expression of 93.3%, 90.7%, and 73.9%, respectively, thus indicating that *MDM2* amplified sarcomas showed a better prognosis. Our present study indicated that intimal sarcomas with an *MDM2* amplification had a better prognosis than non‐amplified undifferentiated sarcomas. These findings are the first to be reported in primary cardiac sarcomas. It will therefore be important going forward, from a prognostic perspective, to distinguish intimal sarcomas with *MDM2* amplifications from UPS, and this may serve as a basis for the application of *MDM2* inhibitors as future therapies.

Our findings revealed that adjuvant treatment in primary cardiac sarcoma significantly improved overall survival, which is consistent with the recent study by Hendriksen et al^27^ showing the beneficial effects of postoperative radiation therapy or chemotherapy on patient survival. Based on the analysis according to tumor histology who treated adjuvant chemotherapy or radiation therapy, angiosarcoma showed survival benefit, but no statistically significant difference was observed in intimal sarcoma, suggesting that histology may play a role in the effectiveness of adjuvant treatment. The accurate diagnosis of intimal sarcoma in high‐grade cardiac sarcoma using MDM2 IHC can have a significant impact on patient treatment and prognosis, and therefore, is important.

One of the limitations of our present study was that only *MDM2* protein expression can be identified by IHC, not *MDM2 gene* amplification. However, in accordance with our previous study,^25^ MDM2 protein expression determined by IHC correlates well with *MDM2* gene amplification revealed by FISH, with a sensitivity of 93% and specificity of 100% (*p* < 0.001). Hence, in diagnosing intimal sarcoma, screening by IHC will be a robust and accurate approach without the need to conduct FISH assays. A second limitation of our current analyses was that the detection of *PDGFRA* amplification among the intimal sarcomas could not be confirmed. However, it is not necessary to perform MDM2 gene amplification specifically for diagnosis or prognostic assessment of the patient. As mentioned earlier, there is a strong correlation between MDM2 gene amplification and MDM2 protein expression. Additionally, it is known that not only MDM2 gene amplification but also CDK4 and PDGFR gene amplification can occur together in intimal sarcoma.^28^ Therefore, if CDK4 IHC is performed along with MDM2 gene amplification testing, the diagnosis should not be challenging. Sai et al. revealed that strong PDGFR expression in pulmonary arterial sarcoma showed long‐term stable disease when treated with Pazopanib.^29^ Therefore, confirming PDGFR expression or amplification seems important in terms of providing targeted therapy for the patient. While it may not be necessary for the diagnosis itself, it can be considered a crucial test for treatment purposes. Instead of performing each individual test separately, utilizing Next Generation Sequencing (NGS) to identify druggable targets all at once could be a time‐saving, cost‐effective, and sample‐conserving approach. Another limitation was that we did not analyze the survival rates over the different time periods included in the study due to the small number of patients. As improvements are made to the various treatment modalities for cardiac sarcoma and new drugs are developed, the survival rate will be expected to improve for the affected patients.^14^ In our study, we have confirmed the important fact that adjuvant treatment increases the survival of patients. Based on this, providing tailored treatment to patients will be much more effective in their care. Firstly, a phase 1/2 clinical trial of an MDM2 inhibitor is currently underway, and positive results have been reported. At the 2022 American Society of Clinical Oncology Annual Meeting (ASCO 2022), preliminary results of a phase 1 trial (NCT03449381) evaluating BI 907828 in patients with advanced solid tumors showed promising outcomes. In patients with DDLPS, partial responses (PRs) or stable disease (SD) were observed in 88.9% of patients, and in patients with well‐differentiated liposarcoma (WDLPS), PRs or SD were observed in 92.9% of patients. The progression‐free survival for patients with liposarcoma was over 10.5 months.^30^ Additionally, Milademetan (RAIN‐32), an oral MDM2 inhibitor, has initiated clinical trials, including a randomized phase 3 trial (MANTRA [RAIN‐3201]; NCT04979442) in patients with unresectable or metastatic DDLPS.^31^ It is anticipated that MDM2 inhibitors will soon receive FDA approval after the success of these tirals. Secondly, phase II clinical trials such as SARC028 and ALLIANCE have shown the potential positive activity of anti‐PD‐L1 therapies in soft‐tissue sarcoma patients.^32^, ^33^ Furthermore, several clinical trials of immune checkpoint inhibitors are ongoing.^34^, ^35^ Lastly, one solution to address the issue of donor shortage in heart transplantation is the development of transplantable organs, such as a human heart, through tissue engineering. Recently, Tan et al^36^ announced the results of creating a functioning heart by seeding differentiated cardiac cells derived from discarded human heart tissue with pluripotent stem cells and cultivating them in vitro for an extended period. While there are still challenges to overcome, this research demonstrates that the day is not far off when it will be possible to create a fully functional heart in vitro using a patient's own cells, minimizing immunological concerns. Unfortunately, Yin et al^3^ have reported that cardiac sarcoma patients diagnosed within a recent decade (2006–2015) did not achieve a better overall survival (*p* = 0.13) compared to cases from earlier decades (1973–2005). As a final limitation, due to the rarity of primary cardiac sarcoma, the sample size included in this study was too small. To overcome this limitation, we hope that multicentric studies with a large cohort will support our research findings.

In conclusion, our study supports the use of adjuvant treatment in primary cardiac sarcoma, as it was associated with a significantly better overall survival rate. Further consideration of tumor histology may be important in determining the optimal use of adjuvant treatment for different types of sarcomas. The reason for emphasizing the MDM2 test is that if MDM2 overexpression is detected in high‐grade sarcoma, it should be classified as intimal sarcoma, which has a better prognosis compared to UPS, thus, accurate diagnosis by MDM2 test is important. In addition, when considering adjuvant treatment, histology should also be taken into consideration as there is no statistically proven survival benefit of adjuvant treatment.

## AUTHOR CONTRIBUTIONS

**Haeyon Cho:** Data curation (equal); formal analysis (equal); investigation (equal); writing – original draft (equal). **In Hye Song:** Data curation (equal); investigation (equal); writing – original draft (equal). **Uiree Jo:** Conceptualization (equal); data curation (equal); formal analysis (equal); investigation (equal). **Ji‐Seon Jeong:** Data curation (equal); formal analysis (equal); methodology (equal). **Hyun Jung Koo:** Data curation (equal); investigation (equal). **Dong Hyun Yang:** Data curation (equal); investigation (equal). **Sung‐Ho Jung:** Data curation (equal); investigation (equal). **Joon Seon Song:** Conceptualization (equal); funding acquisition (equal); resources (equal); supervision (equal); writing – review and editing (equal). **Kyung‐Ja Cho:** Conceptualization (equal); writing – review and editing (equal).

## FUNDING INFORMATION

This study was supported by a grant (2018IL‐0664) from the Asan Institute for Life Sciences, Asan Medical Center, Seoul, Korea. (JSS).

## CONFLICT OF INTEREST STATEMENT

The authors declare no competing interest.

## ETHICS STATEMENT

This study was approved by the Institutional Review Board of Asan Medical Center, Seoul, Republic of Korea, (IRB no. 2016–0180). The requirement for written informed consent was waived by the institutional review board of the Asan Medical Center (IRB no. 2016–0180) due to the retrospective nature of this study. All procedures performed in this study involving human participants were in accordance with the ethical standards of the institutional research committee and the 1964 Declaration of Helsinki and its later amendments.

## Supporting information


**Data S1.** Supporting Information.

**Supplement Table 1**. Patient demographics

## Data Availability

The authors declare that all the other data supporting the findings of this study are available within the article and its supplementary information file and from the corresponding author upon reasonable request. Also, the authors declare that all codes supporting the findings of this study are available from the corresponding author upon reasonable request.
