# Supplementary material for: Primary cardiac sarcomas: A clinicopathologic study in a single institution with 25 years of experience with an emphasis on MDM2 expression and adjuvant therapy for prognosis
Source: Cancer Med. 2023 Jul 3;12(16):16815–28. doi: 10.1002/cam4.6303 (PMC10501235; doi:10.1002/cam4.6303)
Supplement: Supplementary file 1 — Data S1. Supporting Information. [file CAM4-12-16815-s001.docx]

**Supplement methods**

**RNA extraction**

Total RNA was extracted from formalin fixed and paraffin embedded tissue, using the Qiagen RNA extraction kit (Valencia, CA) as directed by the manufacturer, and treated with DNAse I before reverse transcription.

**Reverse transcriptase-polymerase chain reaction (RT-PCR) for the diagnosis of synovial sarcoma**

2 μL of the cDNA reaction with an initial incubation step started at 94°C for 5 minutes. The amplification profile of the PCR consisted of 40 cycles of denaturation at 94°C for 40 seconds, annealing at 55°C for 40 seconds and extension at 72°C for 60 seconds and 10 minutes. PCR amplifications were performed using the following primers: SYT-1(5′-GGATATAGACCAACAC AGCCTGGA-3′), SYT-2(5′-CAGCAGAGGCCTTATGGATATGAC-3′) and SSX (5′-GGGCCAGATGCTTCTG ACACT-3′). The reaction products were subjected to electrophoresis in 2.5% agarose gel and visualized by ethidium bromide staining. The size of PCR products was 97 bp for the SYT-SSX (SYT-SSX1 or SYT-SSX2) fusion gene

**Representative image of synovial sarcoma RT-PCR**


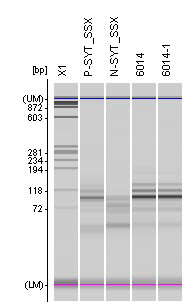


supplement figure 1. The representative image of RT-PCR for synovial sarcoma. The left column represents amplicon ladder. P-SYT-SSX represents positive control and the PCR product are shown in 96 bp. N-SYT-SSX represents negative control. The 6014 and 6014-1 represent the testing sample number. This sample shows the same height of positive control, supporting the presence of translocation for SYT-SSX.
