# Supplementary material for: Primary cardiac sarcomas: A clinicopathologic study in a single institution with 25 years of experience with an emphasis on MDM2 expression and adjuvant therapy for prognosis
Source: Cancer Med. 2023 Jul 3;12(16):16815–28. doi: 10.1002/cam4.6303 (PMC10501235; doi:10.1002/cam4.6303)
Supplement: Supplementary file 2 — Supplement Table 1. Patient demographics [file CAM4-12-16815-s002.docx]

**Supplement Table 1. Patient demographics**

| **Parameters** | **N (n=48, %)** |
| --- | --- |
| **Age (median, years)** | 47 (range, 12-78) |
| **Gender** |  |
| Male | 21 (43.8%) |
| Female | 27 (56.3%) |
| **Histology** |  |
| Angiosarcoma | 23 (47.9%) |
| Intimal sarcoma | 13 (27.1%) |
| UPS | 2 (4.2 %) |
| Others† | 10 (20.8%) |
| **Tumor location** |  |
| Right atrium | 27 (56.3%) |
| Left atrium | 14 (29.2 %) |
| Right ventricle | 5 (10.4 %) |
| Left ventricle | 1 (2.1%) |
| Pericardium | 1 (2.1%) |
| **Surgical treatment** |  |
| Incomplete excision | 33 (68.8 %) |
| Complete excision | 11 (22.9%) |
| Heart transplatation | 4 (8.3%) |
| **Adjuvant Tx** |  |
| No | 11 (22.9%) |
| CTx | 24 (50.0%) |
| RTx | 2 (4.2%) |
| CTx+RTx | 9 (18.9%) |
| **Neoadjuvant Tx** |  |
| Yes | 4 (8.3%) |
| No | 38 (79.2%) |

† Others includes synovial sarcoma, fibrosarcoma, Ewing sarcoma, myxofibrosarcoma and malignant peripheral nerve sheath tumor.

**UPS**, undifferentiated pleomorphic sarcoma; **Tx**, treatment; CTx, chemotherapy; RTx, radiation therapy ;
